# Incremental Prognostic Value of Wearable-Derived Heart Rate and Activity Circadian Features in Tricuspid Regurgitation

**DOI:** 10.1016/j.jacadv.2026.103270

**Published:** 2026-09-23

**Authors:** Valentine Ojonugwa Idakwo, Christian Goelz, Gokce Yumusak, Lukas Stolz, Fabian Scheipl, Jörg Hausleiter, Thomas J. Stocker, Solveig Vieluf

**Affiliations:** a Department of Medicine I, LMU University Hospital, LMU Medizin, Ludwig-Maximilians-Universität München, Munich, Germany; b German Center for Cardiovascular Research (DZHK), Partner Site Munich Heart Alliance, Munich, Germany; c relAI – Konrad Zuse School of Excellence in Reliable AI, Munich, Germany; d Department of Statistics, Ludwig-Maximilian University Munich, Munich, Germany; e Munich Center for Machine Learning, Ludwig-Maximilian University, Munich, Germany

**Keywords:** circadian rhythm, heart failure, remote patient monitoring, tricuspid regurgitation, wearables

**Figure:**
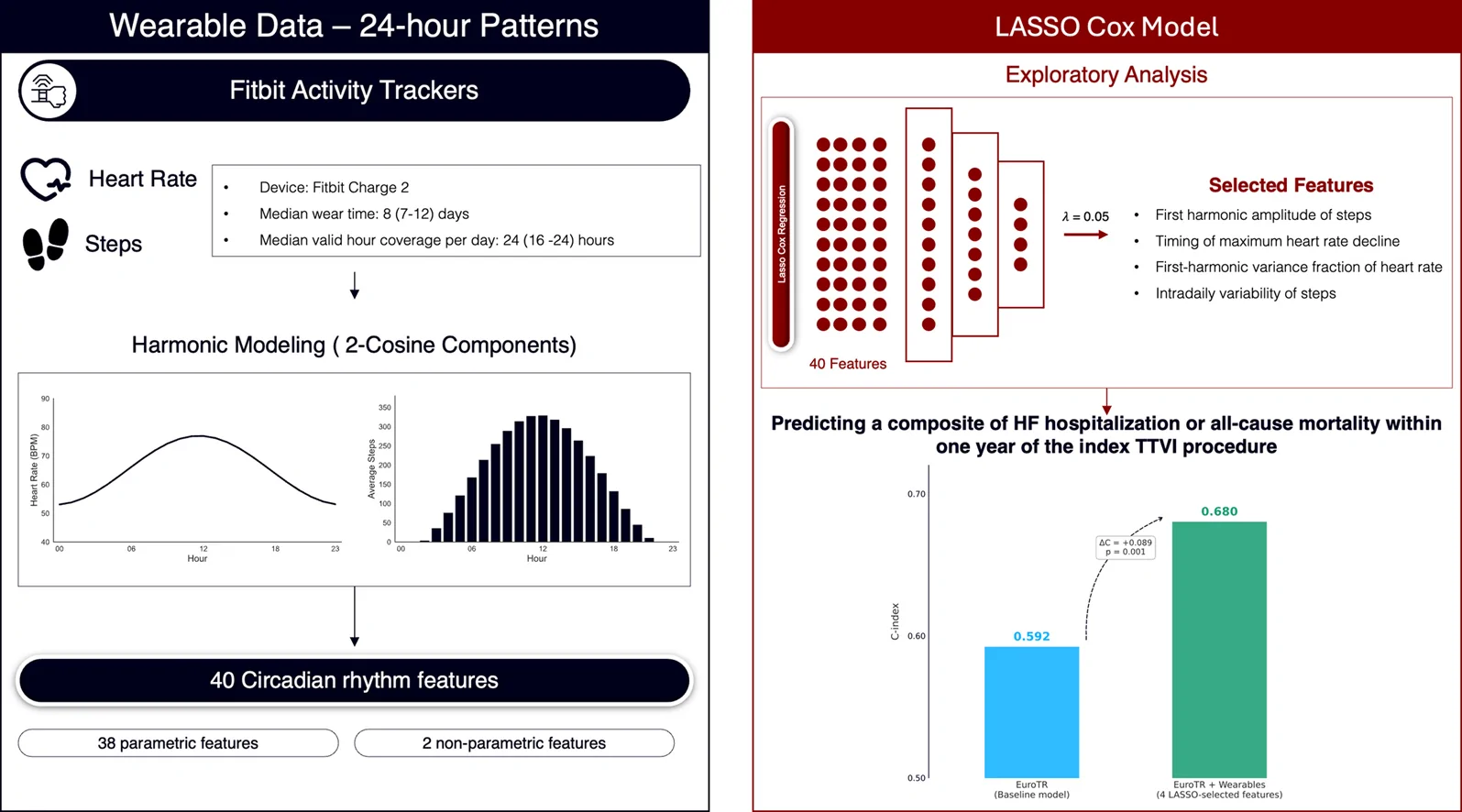

**What is the clinical question being addressed?**

Do wearable-derived 24-hour rhythm features improve prediction of the composite endpoint of 1-year all-cause mortality and heart failure hospitalization beyond the EuroTR score?

**What is the main finding?**

In exploratory, hypothesis-generating analyses, the addition of wearable-derived heart rate and activity features improved prediction of the composite endpoint of 1-year all-cause mortality or heart failure hospitalization beyond the EuroTR score.

Tricuspid regurgitation (TR) in heart failure (HF) is a serious and progressive disease. While transcatheter tricuspid valve interventions (TTVIs) improve quality of life and reduce HF hospitalizations, improved risk stratification is needed to identify at-risk patients.^1^ Current tools rely on static clinical and imaging data, which may miss real-world functional fluctuations. We evaluated whether wearable-derived circadian rhythm features of heart rate and physical activity provide incremental prognostic information beyond the EuroTR Score^2^ for a composite endpoint of 1-year post-TTVI all-cause mortality or HF hospitalization.

We retrospectively analyzed 610 patients with HF and severe TR who underwent TTVI at the LMU University Hospital, Munich (2017-2024). Wearable data were collected using wrist-worn activity trackers (Fitbit Charge 2, Fitbit, Inc). Of 213 patients with baseline Fitbit data, 212 met the minimum density criterion (1 hourly heart rate measurement/day) and were included. Median wear time before TTVI was 8 (7-12) days. The EuroTR score was calculated before the TTVI. The primary endpoint was the composite of HF hospitalization or all-cause mortality within 1 year of the index TTVI procedure. We modeled the 24-hour heart rate and activity profiles using harmonic regression with 24 and 12-hour cosine components, deriving measures of rhythm strength, timing, shape, and rate of change, supplemented by two nonparametric measures (interdaily stability and intradaily variability). We used Cox proportional hazards survival models to assess the incremental prognostic value beyond the EuroTR score. Three prespecified nested models were compared: EuroTR alone, EuroTR plus heart rate circadian amplitude and bathyphase, and EuroTR plus heart rate circadian amplitude, bathyphase, and step-count circadian amplitude, selected a priori for biological relevance to autonomic and cardiovascular regulation.^3^ In an exploratory sensitivity analysis, all 40 candidate wearable features were entered into an L1-penalized (LASSO) Cox regression, retaining features surviving moderate regularization (λ = 0.05, selected a priori from a penalty path of λ = 0.01-1.0). Discrimination was assessed via apparent Harrell’s C-index, corrected for optimism using Harrell’s bootstrap (1,000 resamples), with feature screening, LASSO selection, and refitting repeated within each resample. Incremental value was assessed via the likelihood ratio test and paired bootstrap C-index differences. No adjustment was made for multiple-endpoint or model-family comparisons. Calibration was assessed by regressing observed risk on mean predicted risk across quartiles of predicted probabilities. Given the limited number of observed events (52 composite events; 14 deaths), the LASSO model and mortality analyses are hypothesis-generating; the multiple selection and comparison procedures performed carry an elevated risk of false-positive findings. Reporting followed TRIPOD + AI guidelines.^4^ This study followed the Declaration of Helsinki and was approved by the local ethics committee. All patients provided written informed consent.

Of the 212 included patients, the primary composite endpoint (HF hospitalization or all-cause mortality at 1 year) occurred in 52 (24.5%). The 1-year all-cause mortality rate was 6.6%. Median age was 80.0 (77.0-83.2) years, and 104 (49.1%) participants were female. Baseline TR was moderate in 8 (3.8%), severe in 109 (51.4%), massive in 79 (37.3%), and torrential in 16 (7.5%) patients. EuroTR significantly predicted both 1-year mortality (HR: 1.04; 95% CI: 1.01-1.06; *P* = 0.002) and the composite endpoint (HR: 1.01; 95% CI: 1.00-1.02; *P* = 0.008). For 1-year mortality, EuroTR’s in-sample C-index was 0.745 (corrected: 0.742; 95% CI: 0.641-0.848), consistent with the originally reported C-index of 0.741 (95% CI: 0.699-0.783) in the EuroTR validation cohort. Discrimination was weaker for the composite endpoint (in-sample C-index: 0.592 [corrected: 0.589]; 95% CI: 0.519-0.670). High-risk patients (EuroTR ≥64th percentile, as defined in the original EuroTR derivation study^2^) had significantly lower event-free survival than low-risk patients (log-rank *P* = 0.003).

Adding prespecified circadian features to EuroTR did not significantly improve discrimination for either endpoint. For 1-year mortality, adding heart rate circadian features increased the C-index from 0.745 to 0.770 (corrected: 0.739; ΔC: +0.024; 95% CI: −0.025 to 0.071; *P* = 0.33), with minimal additional gain with step-count amplitude (C-index: 0.771 [corrected: 0.728]; ΔC: +0.025; 95% CI: −0.029 to 0.078; *P =* 0.50). Similarly, for the composite endpoint, the increase was minimal (0.592 → 0.628 [corrected: 0.589 → 0.593]; ΔC: +0.037; 95% CI: −0.028 to 0.096; *P* = 0.21). However, four LASSO-selected features improved composite-endpoint prediction (C-index: 0.680 [corrected: 0.653]; ΔC: +0.089; 95% CI: 0.016-0.166; *P* = 0.001), whereas none were retained for mortality, likely reflecting the limited number of cases (n = 14). In a secondary, stratified refit at the validated 64th-percentile threshold (EuroTR PH violation: Schoenfeld *P* = 0.03), the four features remained jointly significant for the composite endpoint (likelihood ratio test, *P* = 0.001; C-index of 0.657, 95% CI: 0.583-0.740). Reduced first-harmonic step amplitude (blunted day-night oscillation) was independently associated with increased composite-endpoint risk (HR per SD: 1.24; 95% CI: 1.01-1.51; *P* = 0.036). In addition, greater intradaily step variability showed a borderline positive association (HR per SD: 1.40; 95% CI: 0.98-2.00; *P* = 0.067). Higher first-harmonic variance fraction and earlier timing of maximum heart rate decline showed nonsignificant trends toward lower risk of the composite endpoint (HR per SD: 0.81; 95% CI: 0.62-1.05; *P* = 0.105 and HR per SD: 0.80; 95% CI: 0.62-1.03; *P* = 0.085, respectively).

All models showed reasonable calibration, with quartile-based calibration slopes ranging from 1.03 to 1.14 and intercepts from −0.038 to −0.007. The exploratory LASSO-selected feature model achieved the widest separation of predicted risk across quartiles (8%-47%) vs the EuroTR-only model (12%-40%).

We evaluated the prognostic value of wearable-derived circadian features for predicting a combined endpoint of 1-year mortality or HF hospitalization in patients with significant TR undergoing TTVI. Prespecified circadian features (heart rate amplitude, bathyphase, and step amplitude) did not significantly improve discrimination beyond EuroTR. However, in exploratory, data-driven analyses, four LASSO-selected features significantly improved prediction of the composite endpoint beyond the EuroTR score (Figure 1). Specifically, reduced first-harmonic step amplitude and greater intradaily activity variability were associated with increased risk, while later heart rate decline and more regular 24-hour heart rate patterns showed protective trends.

**Figure 1:**
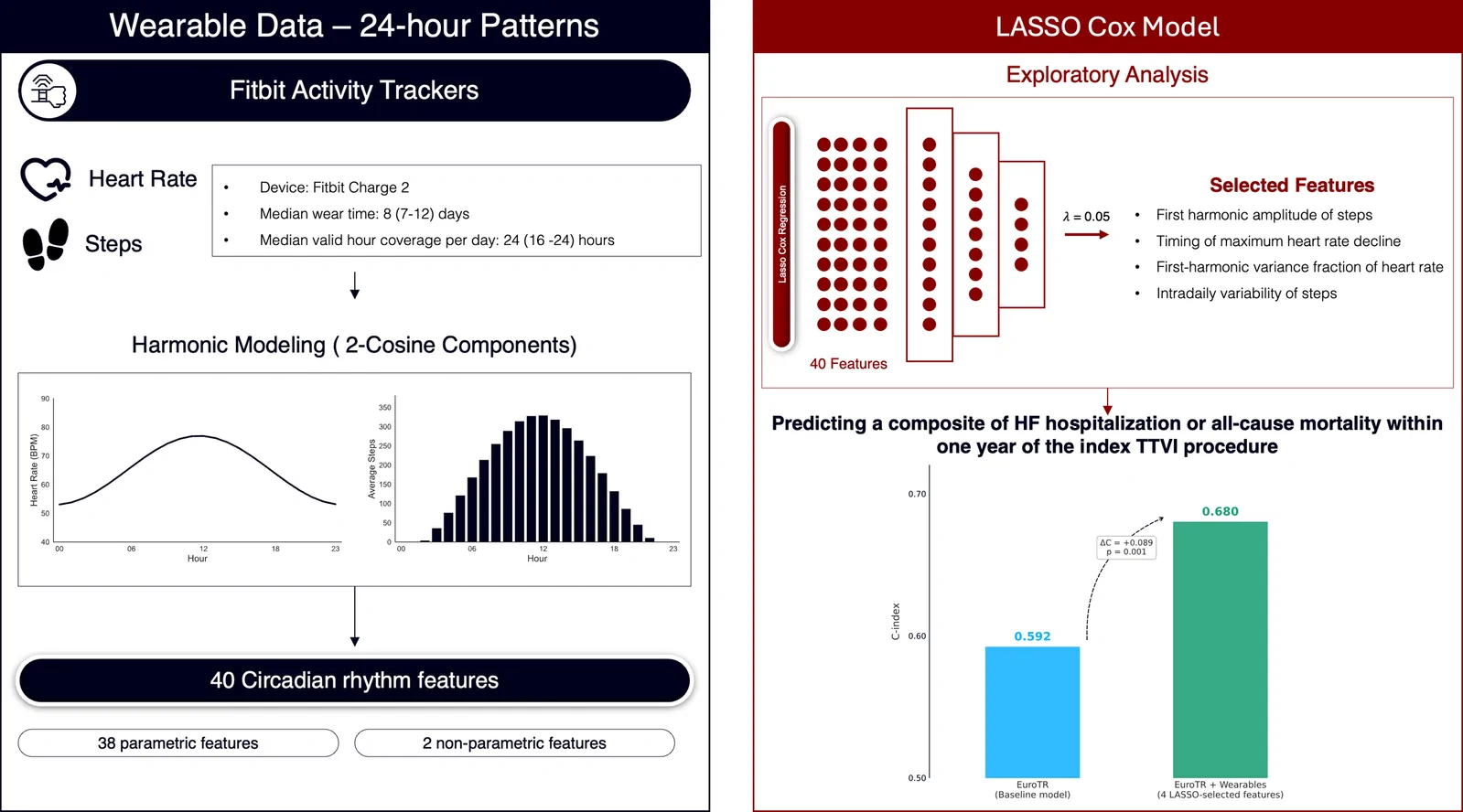
Wearable-Derived Circadian Features for Risk Stratification Beyond EuroTR Continuous heart rate and step-count data from wrist-worn Fitbit devices were modeled using two-harmonic cosine regression to extract 40 circadian rhythm features per patient (left). In exploratory LASSO Cox regression (λ = 0.05), four features were selected: first-harmonic step amplitude, timing of maximum heart rate decline, first-harmonic heart rate variance fraction, and intradaily step variability (right). Adding these features to the EuroTR score improved discrimination for the composite endpoint of 1-year all-cause mortality or HF hospitalization after TTVI, increasing the C-index from 0.592 to 0.680 (ΔC = +0.089; *P* = 0.001). HF = heart failure; TTVI = transcatheter tricuspid valve intervention.

These findings suggest that consumer wearable devices capture aspects of daily functional status invisible during intermittent clinical visits. The differing results for the composite endpoint and mortality are consistent with the notion that continuously recorded wearable-derived patterns are more strongly associated with HF events than with mortality in this cohort.^5^

Several limitations should be acknowledged. First, this was a single-center study with a modest sample size, particularly for mortality events, limiting statistical power. Second, patients with baseline wearable recordings represented a self-selected, less clinically severe subgroup of the overall TTVI cohort, limiting the applicability of these findings to patients able to complete preprocedural wearable monitoring. Third, although internally validated by bootstrap optimism correction, all estimates are derived from a single cohort; external validation and recalibration in an independent cohort are required before clinical application. Fourth, the LASSO analysis searched 40 candidate features against 52 composite events and 14 deaths, below recommended events-per-variable thresholds, further supporting the hypothesis-generating nature of these findings.

In conclusion, prespecified circadian features did not improve predictions beyond the EuroTR score in patients with TR undergoing TTVI. In exploratory analysis, however, wearable-derived features provided incremental, hypothesis-generating prognostic value for 1-year mortality or HF hospitalization, warranting validation in larger cohorts. These findings underscore the potential of consumer wearable technologies to capture relevant physiological signatures and highlight their role in home-based risk stratification and longitudinal monitoring of TR.

## Funding support and author disclosures

This project was supported by a grant from the Deutsche Herzstiftung e.V. (F/24/24). Drs Idakwo, Goelz, and Vieluf are supported by the Hightech Agenda Bavaria. Dr Stocker received research grant support and speaker honoraria from Edwards Lifesciences and speaker honoraria from AstraZeneca. Dr Hausleiter has received speaker honoraria and research support from Abbott Vascular and Edwards Lifesciences. Dr Stolz has received speaker honoraria from Edwards Lifesciences. Dr Vieluf is a co-inventor on unlicensed patent applications and patents related to wearable technology for seizure monitoring and is an unpaid member of an International League Against Epilepsy task force on wearables. Dr Goelz is a co-inventor on unlicensed patent applications and patents related to wearable technology for seizure monitoring. All other authors have reported that they have no relationships relevant to the contents of this paper to disclose.
